# Isabl Platform, a digital biobank for processing multimodal patient data

**DOI:** 10.1186/s12859-020-03879-7

**Published:** 2020-11-30

**Authors:** Juan S. Medina-Martínez, Juan E. Arango-Ossa, Max F. Levine, Yangyu Zhou, Gunes Gundem, Andrew L. Kung, Elli Papaemmanuil

**Affiliations:** 1 Memorial Sloan Kettering Cancer Center, New York, NY USA; 2 Isabl Inc., New York, NY USA

**Keywords:** Data processing, Analysis information management system, Next generation sequencing, Genomics, Image processing, Software engineering, Multimodal data

## Abstract

**Background:**

The widespread adoption of high throughput technologies has democratized data generation. However, data processing in accordance with best practices remains challenging and the data capital often becomes siloed. This presents an opportunity to consolidate data assets into digital biobanks—ecosystems of readily accessible, structured, and annotated datasets that can be dynamically queried and analysed.

**Results:**

We present Isabl, a customizable plug-and-play platform for the processing of multimodal patient-centric data. Isabl's architecture consists of a relational database (Isabl DB), a command line client (Isabl CLI), a RESTful API (Isabl API) and a frontend web application (Isabl Web). Isabl supports automated deployment of user-validated pipelines across the entire data capital. A full audit trail is maintained to secure data provenance, governance and ensuring reproducibility of findings.

**Conclusions:**

As a digital biobank, Isabl supports continuous data utilization and automated meta analyses at scale, and serves as a catalyst for research innovation, new discoveries, and clinical translation.

## Background

Genome profiling represents a critical pillar for clinical, translational, and basic research. With an ever expanding suite of high-throughput technologies [1], the pace at which the scientific community is generating data at scale has rapidly accelerated. This imposes demands for specialized expertise to support data processing and analysis [2]. Importantly, the derivation of novel biological and clinical insights is increasingly reliant upon large and statistically powered datasets, rich metadata annotation (clinical, demographic, treatment, outcome) as well as integration of diverse data modalities generated across samples and patients (i.e. genomic, imaging) [3]. Such high-dimensional data science is now embedded across disciplines, raising significant hopes for the development of artificial intelligence (AI) driven innovation in healthcare and research [3, 4]. However, for this aspiration to fully materialize there is a clear and unmet need for the development of AI-ready data architectures or *digital biobanks.*

Implementation of frameworks that operate in accordance with data processing best practices is important to secure governance and provenance of digital assets, ensure quality control, and deliver reproducible findings. Analysis Information Management Systems (AIMS) [5–9] for Next Generation Sequencing (NGS) data represent integrative software solutions to support the lifecycle of genomics projects [5]. While the democratization of NGS technologies has driven a development boom across data processing software [5, 9–12], only a few AIMS’s exist to support the increasing user-bases of NGS data and none to our knowledge incorporates multimodal data types in a patient or individual centric architecture.

We have developed Isabl, a plug-and-play platform for the processing of individual-centric multimodal data. Isabl is designed to support: (1) management of data assets according to the FAIR [13] principles (Findable, Interoperable, Accessible, Reusable), (2) automated deployment of data processing applications following the DATA [7] reproducibility checklist (Documentation, Automation, Traceability, and Autonomy); and, (3) advanced integrations with institutional information systems across diverse data types (i.e. clinical and biospecimen databases). To support flexible workflows Isabl is built upon a customizable framework, that enables end-users to specify metadata and pipeline implementation. In addition, we present a pipeline development methodology that is guided by the principles of containerization [14], continuous integration, version control, and the separation of analysis and execution logic. Here we provide a framework for the development of *digital biobanks—*patient-centric ecosystems of structured, annotated, and linked data that is readily computable upon, mined, and visualized.

## Implementation

### System overview

#### Platform architecture

**Fig. 1:**
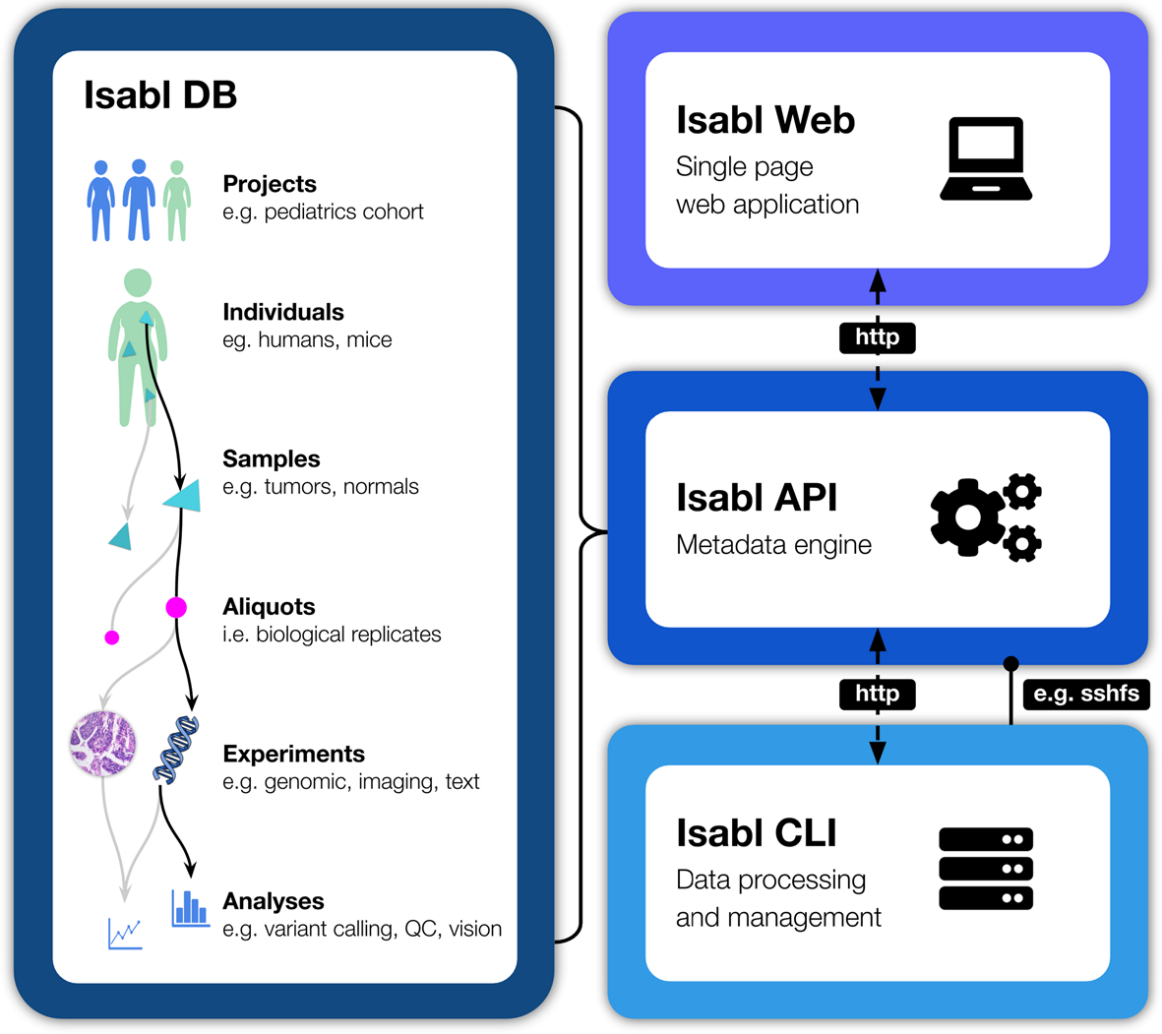
Schematic representation of Isabl's microservice architecture. Isabl DB provides a patient centric relational model for the integration of multimodal data types (i.e., genomic, imaging) and their corresponding relationships (individual, sample, aliquot, experiment, analyses). Isabl Web facilitates visualization of results and metadata in a single page application. Isabl API powers the linkage to other institutional information systems and is agnostic to data storage technologies and computing environments, ensuring metadata is accessible even when the data is no longer available (FAIR A2). Isabl CLI is a Command Line Client used to process and manage digital assets across computing paradigms (i.e. cloud, cluster). Arrow connectors indicate database relationships between Isabl schemas, dashed lines indicate metadata transfer through the internet, solid line indicates a data link between the data lake and the web server (e.g. sshfs, s3fs, https)

Isabl is composed of four main microservices [15] (Fig. 1): (1) Isabl DB, an individual-centric database designed to track patients, samples, data, and results; (2) Isabl API, a RESTful API used to support authentication, interoperability, and integration with data processing environments and enterprise systems (e.g. clinical databases, visualization platforms; FAIR A1 [13]); (3) Isabl CLI, a Command Line Interface for managing and processing digital assets in a scalable data lake (i.e., genomic, imaging); and (4) Isabl Web, a frontend single page web application for data interrogation (for further technical details please refer to Isabl's documentation https://docs.isabl.io/quick-start; https://github.com/isabl-io/docs).

#### Data model

**Fig. 2:**
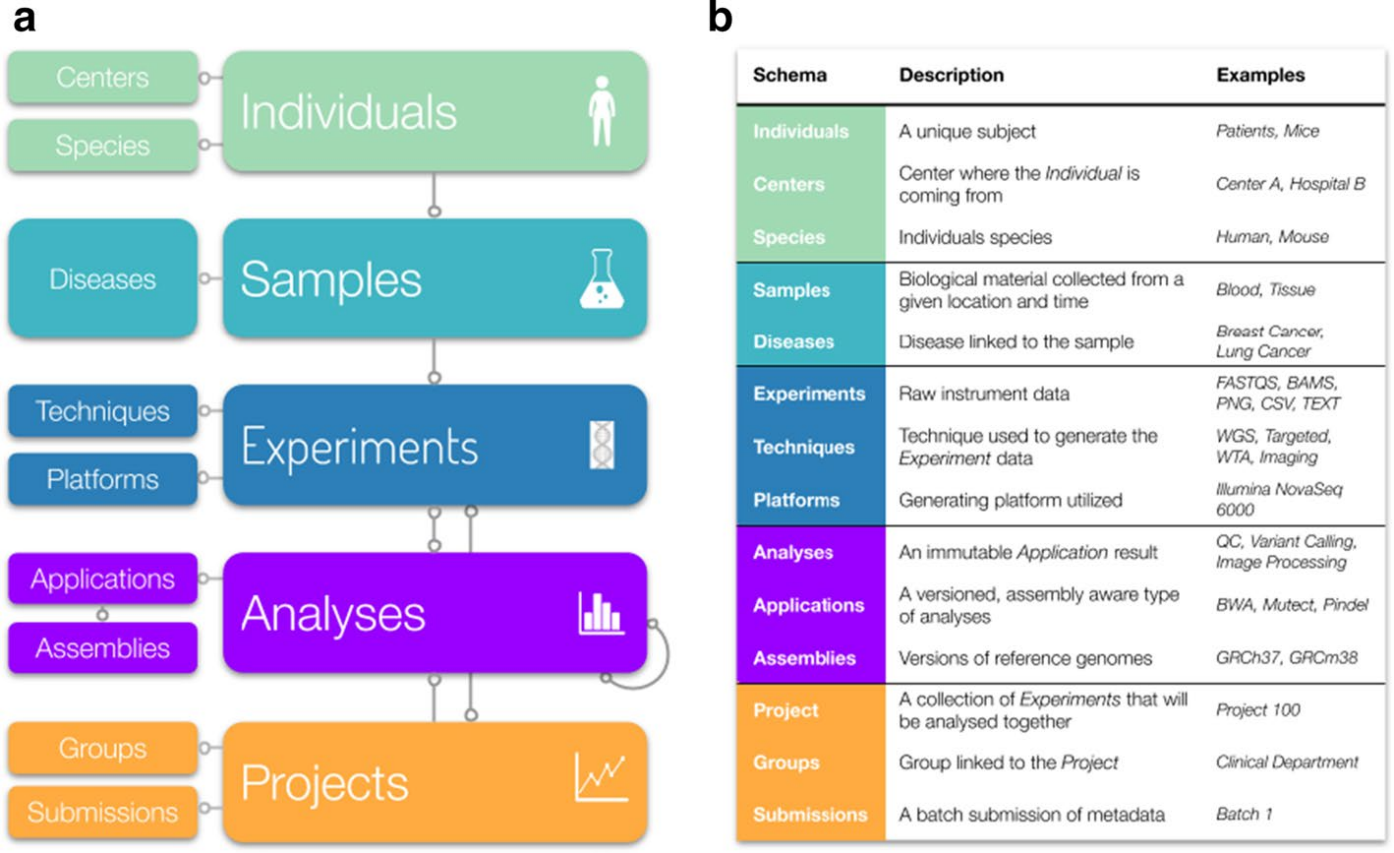
Isabl's relational model maps workflows for data provenance (e.g. Individuals, Samples, Experiments), processing (e.g. Applications, Analyses), and governance (e.g. Projects, Users). **a** An individual-centric model facilitates the tracking of analyses conducted on experimental data obtained from related samples. Analyses are results of analytical workflows, or applications. Experiments are analyzed together and grouped in projects. Additionally, schemas to track metadata for diseases, experimental techniques, data generation platforms, and analyses cohorts are also provided. Lines with one circle represent foreing keys, whilst lines with two circles represent many to many relationships. **b** A brief description of these schemas with examples

Isabl DB maps workflows for data provenance, processing, and governance (Fig. 2; FAIR R1 [13]). Metadata is captured across the following 5 thematic categories: (1) patient attributes; (2) samples, as biological material collected at a given time; (3) data properties including experimental technique, platform technology, and related parameters; (4) analytical workflows to account for a complete audit trail of versioned algorithms, related execution parameters, reference files, status tracking, and results deposition; (5) data governance information across projects and stakeholders (Additional file 1: Fig. S1; FAIR F2 [13]). All database records are assigned a globally unique and persistent identifier (UUID; FAIR F1 [13]), whilst individuals, samples, and experiments are further annotated with a customizable human friendly identifier (Additional file 1: Fig. S2). All metadata stored in Isabl DB is version controlled, all changes are recorded and previous states can be recovered. Management of phenotypic data such as disease ontology can be facilitated in three ways. Firstly, the disease schema can be customized with additional fields in agreement to end-user requirements. Secondly, ontologies from established databases such as OncoTree, (http://oncotree.mskcc.org) can be integrated (i.e. https://docs.isabl.io/data-model#sync-diseases-with-onco-tree). Lastly, proprietary schemas from institutional databases (i.e. ontologies implemented in local electronic medical records) can also be incorporated, thus allowing for direct linkage between results and related metadata at an institutional level.

## Results

### Life cycle of bioinformatic operations

Isabl operations are organized in a three step process: (1) project initiation and metadata registration; (2) automated data import and processing; and, (3) results retrieval for analyses.

#### Projects and metadata registration

At project initiation, users specify a title, study description, and define stakeholders using Isabl Web. Individuals, samples and related experiments are registered through web forms, Excel batch submissions, or automated HTTP requests. Validation rules are enforced to ensure content quality, while account permissions and user roles guide data governance (project creation, edit, and data queries; see https://docs.isabl.io/production-deployment#multiuser-setup). To prevent dangling information, records can't be deleted if they are associated with other instances (e.g. a sample can't be removed if it has linked experiments). Furthermore, all database schemas can be extended with custom fields in order to address end-user metadata requirements.

**Fig. 3:**
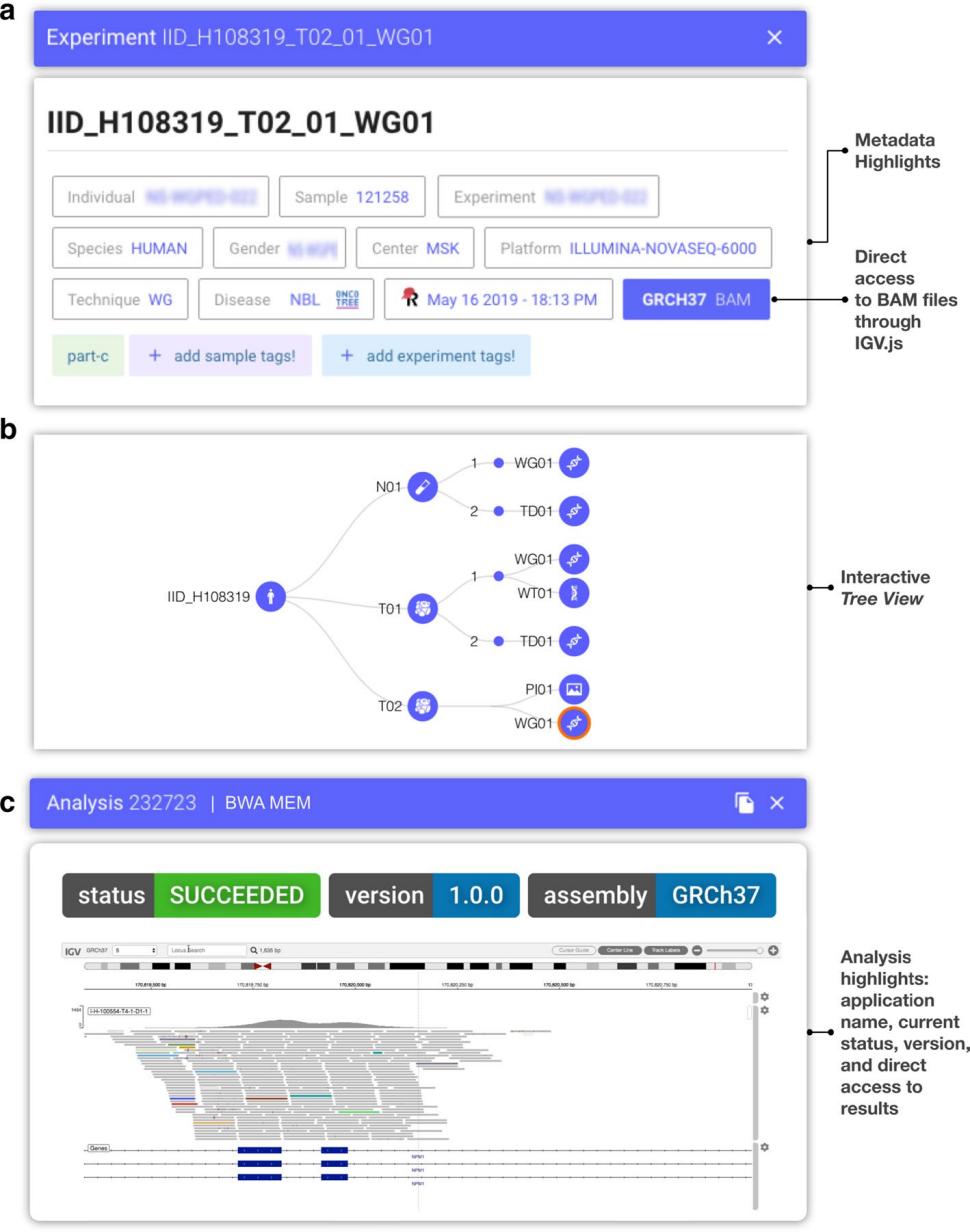
Isabl Web is a Single Page Application (SPA) organized in interactive panels (https://demo.isabl.io). **a** Example of sample level metadata, to include sample ID, corresponding individual ID, experimental ID, species, gender, center, data generating platform, experimental technique, disease state at the time of sampling, institutional database integrations (i.e. RedCap) and version of corresponding data genome assembly. Metadata fields are flexible and customizable. **b** Tree view representation of an individual assets (samples, aliquots, experiments). Users can dynamically explore metadata by clicking the different nodes (i.e. from samples, to experiments, to all available analyses under any node). **c** The Analysis Panel indicates execution status, version, run time, storage usage, linked experiments and offers quick access to a selected set of results (e.g. BAM files with https://github.com/igvteam/igv.js, images, log files, tables)

Once information is registered, users can interrogate the entire digital real estate using Isabl Web. A single page portal is populated with interactive panels that become available as new information is requested (Fig. 3; https://demo.isabl.io). Tables directly wired to Isabl API, provide searching, filtering, and ordering capabilities across different schemas and are available throughout the application (e.g. Additional file 1: Fig. S3; FAIR F4).

Detail views are retrieved by clicking on any hyper-linked identifier within these tables. The project detail panel caters a birds-eye view across all analyses and experiments pertaining to a study (Additional file 1: Fig. S4). Similarly, the samples view provides an interactive, patient-centric, *tree* visualization that enables instant access to all assets generated on a given individual (Fig. 3a, b; Additional file 1: Fig. S5). Dashboards to explore metadata and access results are also provided (Additional file 1: Fig. S6).

#### Data import and registration

After metadata registration, the next step for an Isabl project is data import. Isabl CLI explores data deposition directories (i.e. sequencing core, data drives) identifying multimodal digital assets (i.e. genomic, imaging) relating to specific experiments and imports them into a scalable data directory (move or symlink; Additional file 1: Fig. S7). This process ensures that the link between data and metadata is stored in Isabl DB. Upon import, access permissions are configured and data related attributes are stored in the database (e.g. checksums, usage, location). Import status is updated in Isabl DB and displayed in Isabl Web.

In addition to data imported for analyses, Isabl CLI also supports the registration of auxiliary assets such as an assembly reference genomes, techniques reference data (e.g. BED files), and post-processing files (i.e. data relating to control cohorts). To secure data integrity, import operations and data ownership are limited to a single *admin user* (e.g. a shared Linux account managed by Isabl administrators). Importantly, import logic for data and auxiliary files is entirely customizable and can be tailored to end-user requirements (i.e. cloud storage).

Out of the box, Isabl CLI operates on local file systems using traditional unix commands such as mv, ln, cp, and rsync. Nevertheless, the Isabl data lake can be stored in cloud buckets like Amazon S3 (https://aws.amazon.com/s3), Google Storage Buckets (https://cloud.google.com/storage), or Azure Blobs (https://azure.microsoft.com/services/storage/blobs). Mechanisms to push and pull data to the cloud must be implemented by the user, although there are automated solutions such as Amazon FSx for Lustre (https://aws.amazon.com/fsx/lustre). When data is stored in the cloud, Isabl Web can be configured to retrieve and display results from these providers. Importantly, Isabl can compute on data located in a local file system, cloud based solutions or hybrid (local and cloud).

#### Deploying data processing tools at scale with Isabl applications

**Fig. 4:**
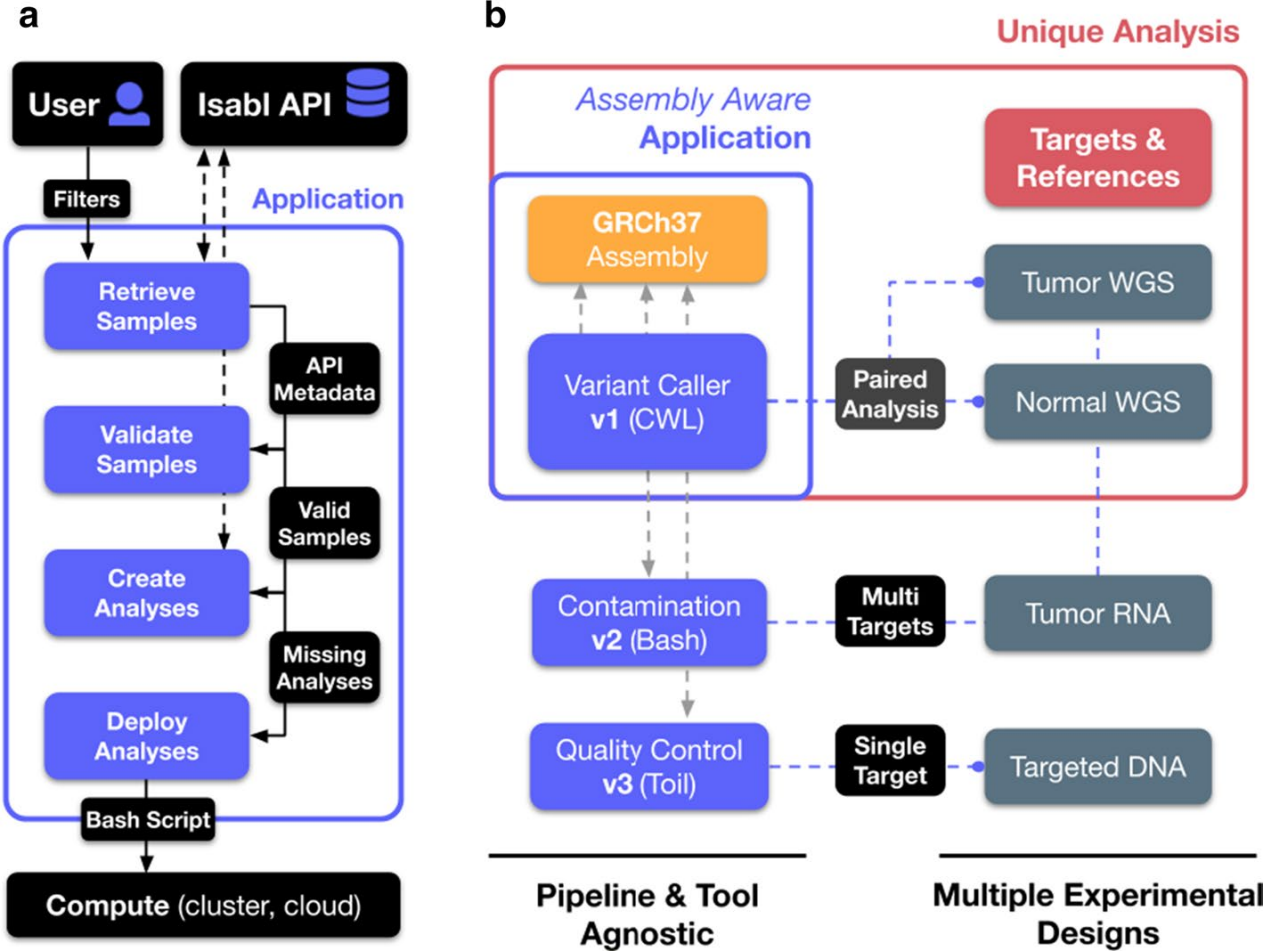
Isabl applications enable systematic processing of experimental data. **a** Guided by metadata, Isabl applications construct, validate, and deploy computing commands across experiments. Applications differ from Workflow Management Systems in that they don't *execute* the analytical logic but *construct* and *submit* a command. **b** Isabl applications can be *assembly aware*, this means that they can be versioned not only as a function of their name, but also as a function of the genome assembly they are configured for. This is important because NGS results are comparable when produced with the same genome version. The unique combination of *targets* and *references*, such as tumor-normal pairs, results in *analyses*. The figure panel illustrates applications with different experimental designs, such as paired analyses, multi-targets, single-target, etc. Importantly, applications are agnostic to the underlying tool or pipeline being executed

Isabl is a horizontally integrated digital biobank onto which existing or bespoke analytical applications can be docked and integrated in a way that confers sample-centric traceability to the analytical results. Upon data import, Isabl applications enable standardized deployment of data processing pipelines with a Software Development Kit (SDK; Fig. 4). Guided by experimental metadata in Isabl DB, applications construct, validate, and deploy execution commands into a compute environment of choice (e.g. local, cluster, cloud; Fig. 4a). Isabl applications are defined using python classes (Additional file 1: Fig. S8).

For example, variant calling applications will tailor execution parameters and reference datasets given the nature of the data (i.e. targeted gene sequencing, whole genome sequencing, etc.). Application results are stored as *analyses* (Fig. 4b)*.* Each *analysis* is linked to results files and specific execution parameters. Analyses can compute on data for one or more *targets* and *references* experiments (e.g. single-target, tumor-normal pairs, target vs. pool of normals, etc.). Furthermore, analyses can also track numeric, Boolean, and text results using a PostgreSQL JSON Field. To warrant a full audit trail of results provenance and foster reproducibility, Isabl stores all analyses configurations (parameters, reference datasets, tool versions, etc.).

Upon completion of an analytical workflow, ownership of output files is automatically transferred to the admin user and write permissions are removed (see https://docs.isabl.io/writing-applications#applications-run-by-multiple-users). Once implemented, applications can be deployed system wide, on an entire project, or any subset of experiments in the database. A user-defined selection of results can be accessed through Isabl Web, which also indicates execution status, version, run time, storage usage, and linked experiments (Fig. 3c; Additional file 1: Fig. S3). If an analysis has already been executed, the system will prevent it's resubmission to minimize computing usage and prevent duplication.

#### Operational automations

To automate downstream analyses Isabl applications define logic to combine results at a project or individual level (Additional file 1: Fig. S9). For example, quality control reports, variant calls, or any other kind of result are merged within a single report (for each result type). The merge operation, at the project *or* individual level, is triggered automatically and runs only when required (i.e. not executed if other *to-be-merged* analyses are ongoing). Aggregated outputs are dynamically updated as new experiments are processed by the application. All auto-merge analyses are versioned and stored in Isabl DB.

Isabl CLI facilitates automations using *signals*, python functions triggered on status changes to execute subsequent tasks (Additional file 1: Fig. S9). For instance, a signal can be configured to deploy quality control applications upon data import. At QC success, another signal could deploy a complete suite of applications tailored to the nature of the experimental data. In case of automation failure, Isabl will send notifications to engineers via email, with error logs and instructions on how to restart the automation. Furthermore, Isabl API is equipped with an asynchronous tasks functionality useful to schedule backend work. For example, a task can be configured to sync metadata from institutional systems every 2 h.

#### Data access and results retrieval

Users can retrieve results using three main mechanisms: (1) visualization through Isabl Web; (2) programmatic data access with Isabl CLI; and, (3) direct data lake access (https://docs.isabl.io/retrieve-data). For each analysis, job execution status (i.e. pending, in progress, complete), as well as a defined list of results can be directly accessed through Isabl Web (with support for strings, numbers, text files, images, PDF, BAM, FASTA, VCF, PNG, HTML, amongst others; Additional file 1: Fig. S3). Isabl Web access to NGS data is further enabled using IGV.js (https://github.com/igvteam/igv.js; Fig. 3c). Additionally, Isabl CLI represents a programmatic means of entry to the entire data capital. A suite of command line utilities for metadata, data, and results retrieval is readily available. For example, queries can be constructed to identify samples of interest matching a range of attributes (i.e. patients, samples, analyses metadata) and retrieve specified results files (e.g. VCF files).

The codebase powering Isabl's client can be imported as a python package fostering systematic administration of data and analyses. For example, an analyst can import the SDK into a Jupyter [16] notebook to automatically access versioned algorithmic output for downstream post-processing, ensuring a full audit trail of data provenance from raw data to analysis and post-processing results. Moreover, Isabl CLI automatically creates and maintains easily accessible project directories with symbolic links pointing to all data and results, thus allowing access independently from the RESTful API (Additional file 1: Fig. S7c).

#### Integration of analytical applications into Isabl

Isabl as a bioinformatics framework is completely agnostic to bioinformatics pipelines and does not include pre-built applications (e.g. variant callers such as Pindel [17], Strelka [18]) or Workflow Management Systems (WMS; e.g. Bpipe [19], Toil [20]). Nevertheless, end-users can package, install, and deploy applications of choice in accordance with their data and operational requirements (e.g. https://github.com/isabl-io/demo). This enables full leverage of Isabl functionality while maintaining complete independence and flexibility in analytical workflows.

To facilitate seamless integration and rapid iteration of data processing pipelines into Isabl, we developed Toil Container and Cookiecutter Toil (Additional file 1: Fig. S10). Cookiecutter Toil (https://github.com/papaemmelab/cookiecutter-toil) is a templating utility that creates tools or pipelines with built-in software development best practices (i.e. version control, containerization, cloud testing, packaging, documentation; Additional file 1: Fig. S10a). On the other hand, Toil Container (https://github.com/papaemmelab/toil-container) enables Toil [20] class-based [10] pipelines to perform containerized system calls with both Docker and Singularity [21] without source code changes (Additional file 1: Fig. S10b). Toil Container ensures that analytical logic remains independent of execution logic by keeping pipelines agnostic to containerization technology or compute environment (e.g. an application can run using Docker in the cloud or Singularity in LSF; Additional file 1: Fig. S10c).

#### User roles and permissions

There are two levels to Isabl data access: interaction with metadata, and interaction with data.

Metadata. Users can create, retrieve, update, and delete metadata using Isabl Web and Isabl API. In order to manage these interactions, Isabl relies on Django Permissions (https://docs.djangoproject.com/en/3.1/topics/auth/default/#permissions-and-authorization). By assigning users to groups, the Isabl administrator can manage the actions granted towards different resources. Isabl offers 3 main roles: (1) Managers are users who can register samples, (2) analysts can run analyses, and (3) engineers can do both, register samples and run analyses. These roles are optional and customizable. Permissions can also be modified to each user specifically.

Data. The Isabl data lake can reside in the cloud or in a local file system. Access to these resources is not managed by Isabl but by a system administrator (i.e. Unix, Cloud). Users that have access to the data lake can execute applications if they have the right metadata permissions (e.g. create and update analyses). Once data is imported and analyses are finished, Isabl removes write permissions to prevent accidental deletion of data. Permissions to download and access data through Isabl Web are managed using Django Permissions.

### Case studies

We charted the end-to-end processes of bioinformatic operations and designed Isabl to address the major challenges in production-grade computational workflows. This includes the disruption of data silos, flexible integration to metadata sources, dynamic access and visualization of data, version control, audit trail, data harmonization, scalability, automation of analytical workflows and resource management (personnel as well as compute). We showcase how Isabl address these issues with the following case studies:

#### Case study 1: scalability and audit trail

**Fig. 5:**
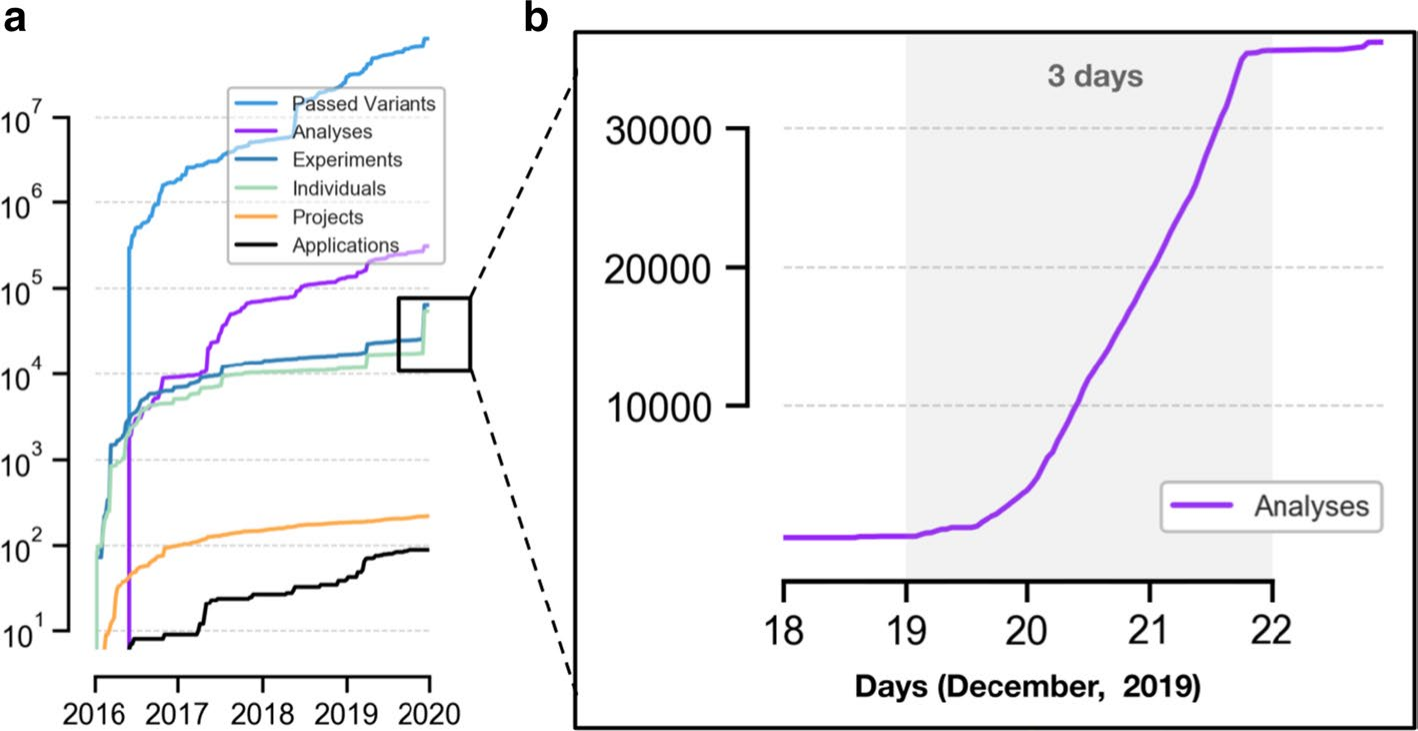
Isabl fosters autonomy, automation, audit trail, and scalable deployment of data processing tools in a system-wide approach. **a** Panel showcases exponential increase in data generation (colored lines indicate categories for registered applications, projects, individuals, experiments, and analyses output). **b** Isabl facilitated the registration and processing of + 35K patients from the MSK-IMPACT cohort using a novel tool. Metadata was ingested with Isabl API in less than an hour, whilst + 35K analyses were submitted with a single command and processed in three days

Isabl has served as the bioinformatics backbone in our center, allowing us to scale up and compute upon data from 60K patients, organized in 200 independent projects. Isabl has supported the deployment of 300K analyses linked to 90 different data processing applications operating on + 300 TB of data—all in a versioned controlled data lake (Fig. 5a) [22–30]. Our Isabl instance maintains a real time audit trail of each step in the data generation process (Additional file 1: Video 1). Results and related metadata are accessible and visualized through Isabl CLI and Isabl Web. Figure 3a indicates the sustained growth in data footprint across time which by leveraging Isabl automations did not impose further demands on personnel.

#### Case study 2: meta analyses, data harmonization, and bugs correction

Meta analyses of existing data sets represent a powerful means to derive new insights. Datasets may be combined to improve statistical power or new algorithms can be executed across projects for novel readouts. For example, Isabl facilitated the fast registration and processing of + 35K patients from the MSK-IMPACT [31] cohort using a novel copy number analysis tool.

Samples metadata was ingested with Isabl API in less than an hour. Subsequently, the deployment of the new tool involved a two step process: (1) application registration; and (2) execution across samples that matched a specific criteria (i.e. targeted sequencing technique equals IMPACT [31]). More than 35K analyses were submitted with a single command and processed in 3 days with a + 5K cpu HPC cluster (Fig. 5b). Resulting output files were harmonized (same version) and organized under a specified project directory.

Similarly, these principles apply to error correction in analytical workflows. Upon discovery of an error or “bug”, Isabl enables the identification of all affected experimental data, re-execution of analyses with a corrected application, and identification of all relevant stakeholders for notification of data status. The pre-existing analyses are transferred to a time-stamped legacy directory. During results retrieval end-users have automatic access to the latest version of each analyses run, but if desired, can retrieve older analyses files from the legacy directory.

#### Case study 3: automation of analytical workflows

**Fig. 6:**
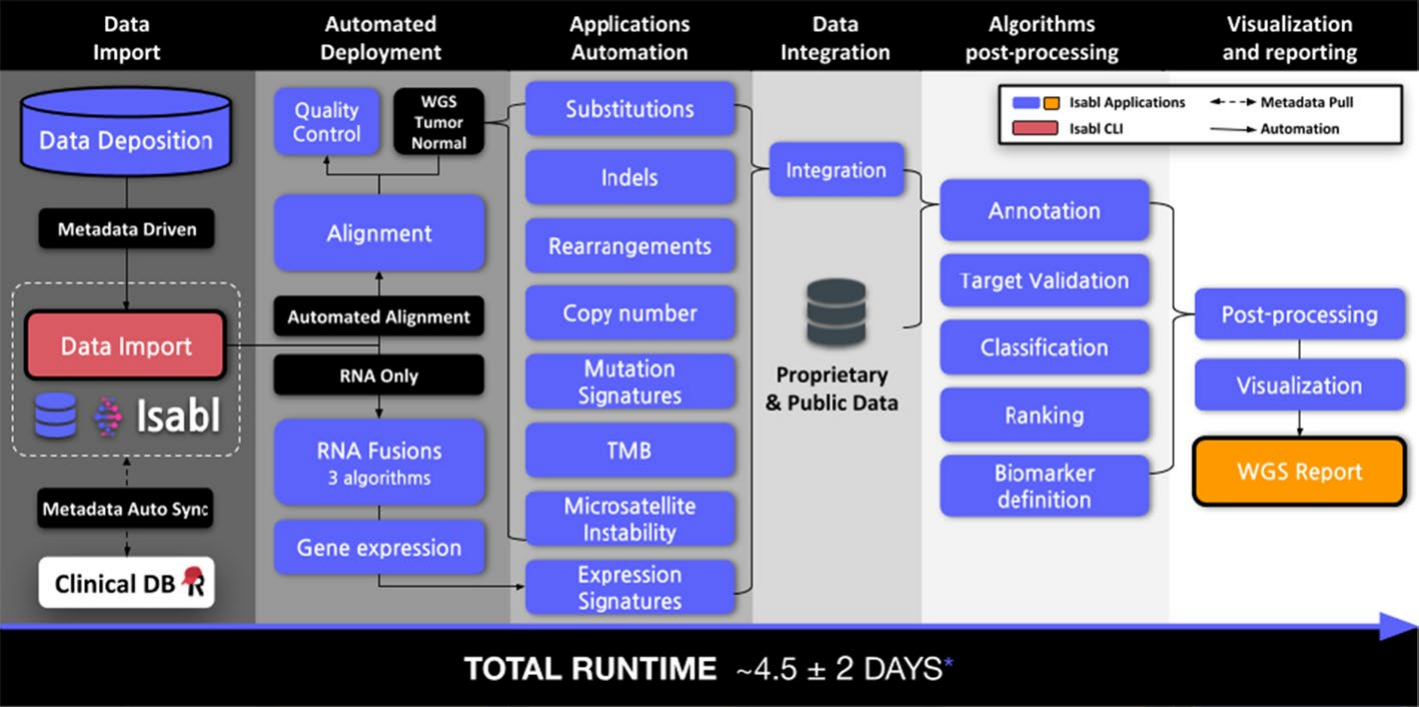
Isabl supports the implementation of production-ready workflows. The *no-click genome* has completed reports at a rate of 4.5 ± 2 days / report (mean ± standard deviation; n = 20; mean depth coverage 80 ± 20) using a 3000-cores High Performance Computing multi-user cluster. Processing duration is primarily driven by the longest-running application at each parallel block as well as compute availability (i.e. cluster congestion)

Isabl was used to implement an automated production-grade workflow for whole genome (WGS) and RNA analysis, executing > 30 independent algorithms automatedly (Fig. 6). Briefly, Isabl CLI and institutional API integrations facilitated the registration of FASTQ files from a sequencing core. Upon import, Isabl automations were used to deploy data processing applications (e.g. alignment, gene counts). Intermediate applications were subsequently executed as prior dependencies were satisfied (e.g. quality-control, variant calling). Last, derivation of summary statistics such as microsatellite instability [32] and homologous DNA recombination scores [33] that depend on primary data extraction (i.e. indels) were executed. Select data was embedded in a patient-centric report accessible through Isabl Web. Termed as the *no-click genome*, the entire process is executed with no manual intervention. In our center, these automations have enabled the discovery of novel diagnostic and therapy informing biomarkers within clinically relevant timeframes [24, 26].

#### Case study 4: multimodal data integration

Whilst Isabl was primarily designed for use cases derived from sequencing data, both platform and analysis paradigms make no assumptions about the nature of the data being registered. For a given individual, sequencing data as well as pathology data can be linked to specific samples [34] (Additional file 1: Fig. S2). The same is true for analysis applications, for example a tiling preprocessing step [35] could be productionized for new pathology images for a biopsy for which whole genome sequencing data is also produced. Analysis output files from image and whole genome sequencing variant calls are linked for a given individual. In this way, Isabl can facilitate the integration of diverse data modalities for downstream correlative analyses, which represents an area of increasing research focus.

### Comparison to other platforms

Beyond Isabl, we have identified four published open source AIMS, (1) the Genome Modeling System (GMS) [5], (2) SeqWare [36], (3) QuickNGS [6] and (4) HTS-flow [9]. Table 1 presents how these platforms compare across five main topics: Metadata Capabilities, Assets Management, Operational Automations, Results Accessibility, and Codebase Status. Although related, the One Touch Pipeline (OTP) [8], SevenBridges [37], FireCloud [38], and other service-based approaches are not considered here as their underlying infrastructures are private. We consider that Omics Pipe [39], Chipster [40], and GenePattern [41] are tools rather than AIMS frameworks. However, we recommend reviewing previous comparisons conducted by Ressinger [8], Bianchi [9], and Wagle [6].

**Table 1:** Five open source Analysis Information Management Systems (AIMS) compared across 5 categories: (1) metadata capabilities, (2) support for assets management, (3) features for systematic data processing, (4) mechanisms for results retrieval, and (5) availability and codebase status

Challenge	Feature	Isabl	GMS	SeqWare	QuickNGS	HTS-flow
Metadata capabilities	Metadata infrastructure	Relational database, RESTful API + Swagger docs	Relational database	Relational database, RESTful API + online docs	Relational database	Relational database
	Data model (ID system)	Individuals Samples Experiments (See Fig. 1)	Individuals Samples Experiments (UUIDs)	Individuals Samples Experiments (User Defined)	Experiments (User Defined)	Experiments (User Defined)
	Metadata ingestion	Excel batch, Web Forms, REST API	Command line Client	Web Form, CLI batch	CSV batch	SMITH LIMS integration
Assets management	Data import	CLI batch	CLI one by one	CLI batch and web	Manual symlink	LIMS integration
	Reference data import	Genomes, BED files, Arbitrary resources	Genomes, variation lists, Ensembl	–	Download scripts for public databases	Genomes download
	Data multimodality	✓	–	–	–	–
	Data organization	Hash-based directory structure	UUID based directory structure	S3 buckets, user defined locations	–	–
Data processing	Apps included	–	✓	✓	✓	✓
	New Apps registration	Python class	Perl components	Java components	Shell scripts	R modules
	WMS (Deployment Support)	WMS Agnostic (Local, LSF)*	Custom WMS (LSF, OpenLava)	Pegasus, Oozie (SGE, AWS)	Shell scripting (SLURM)	Custom WMS (SGE)
	Operational automations	Signals, Project Level auto-merge	–	–	CRON Jobs	–
Results accessibility	Software development kit	Python package	Perl library	–	–	–
	Data provisioning	CLI, file system, download	CLI, file system	CLI, file system, download	Download	CLI, file system, R objects
	Purpose of user interface	Metadata search and ingestion, status monitoring, results access	Metadata search, status monitoring, results access	Metadata search and ingestion, status monitoring	Metadata search, end-user access to results	Metadata search, apps deployment and configuration
Availability and codebase quality	Availability	Docker compose, PyPi (300 MB)	Vagrant VM (200 GB)	Vagrant VM (2 GB)	Manual Install (4 MB)	Manual Install (1 MB)
	Last Commit (Github Stars)	2019	2015 (65)	2016 (26)	Last release 2016	2016 (1)
	Continuous integration	✓	–	✓	–	–
	Docs status	✓	✓	✓	✓	✓
	Programming Languages	Python, Vue, Javascript	Perl, Ruby	Java, JavaScript	Bash, PHP	PHP, R, JavaScript

Upon consideration of the comparison outlined in Table 1 and Additional file 1: Notes 1, Isabl's main differentiators are: (1) integration of a "RESTful API first" approach, (2) support for multimodal data, (3) an implementation agnostic to specific pipelines, workflow management systems, and storage and compute architectures, and 4. it's “plug and play” deployability and extensive documentation. Note that independently these features might not be unique to Isabl, yet the consolidation of all of these features into a single platform is. Importantly, Isabl does not provide integrations to LIMS systems out of the box, and deployment to cloud storage and compute systems require adaptation to the linked architectures.

To showcase Isabl's functionality we developed “10 min to Isabl” (https://docs.isabl.io/quick-start), a tutorial that guides end-users with a personal computer through platform installation, project registration, data import, application execution, and results retrieval.

## Discussion

The collective resources and funding required to support biospecimen collection and data generation in research is formidable. These efforts culminate in data that are mined to answer fundamental questions about human development, population attributes, disease biology and clinical decision support. Whilst sample collections are finite, the data capital if accessible in computable format can be leveraged across time. In the present study we propose the development of digital biobanks as companion infrastructures to support dynamic data access, processing and visualization of the growing data capital in research and healthcare.

To this end, we developed Isabl to support end-to-end bioinformatics operations. We showcase that with Isabl, real world challenges in computational biology, such as quality and version control, analysis audit trails, error correction, scalability, automation, and meta analyses can be readily addressed. To reduce the adoption barrier, the database schema can be customized and analysis tools can be added as *Applications* per end user specifications. To facilitate integration of analytical pipelines in accordance with best practices we further developed and made available Toil Container and Cookiecutter Toil. These templating utilities can be extended to include analyses pipelines for any data modality (NGS, single cell, imaging, etc.). Lastly, to position Isabl as a platform that facilitates and automates large scope institutional initiatives, we have developed a fully documented RESTful API and CLI for integration with biospecimens databases, clinical resources, visualization platforms, sequencing cores, and laboratory information management systems. Although Isabl adheres to the FAIR principles to a great extent, we recognize that the platform could adopt a standardized ontology like FHIR (https://www.hl7.org/fhir/) in the future.

From a strategic and operational perspective, implementation of computable digital biobanks is set to minimize costs by efficiently managing compute resources, reducing time to analyses and importantly demands for hands on operator time to process data. These automations at the same time maximize data deliverables, utilization of the data capital and reproducibility of findings. With the increasing aspiration to develop AI-driven approaches in healthcare and research, we showcase that the development of digital biobanks as AI-ready infrastructures will represent critical catalysts for research innovation, new discoveries, and clinical translation.

## Availability and requirements

Project name: Isabl Platform

Project home page: https://github.com/isabl-io

Operating system(s): platform independent

Programming language: Python, Javascript

Other requirements: Docker Compose

Licence: ad hoc license, free for academic and non-profit institutions

Any restrictions to use by non-academics: licence needed

## Methods

### Architecture and codebase

Isabl architecture is built upon separate codebases, which are loosely coupled and can be deployed independently in a plug-and-play fashion. For example, Isabl Web services only dependency is Docker Compose (https://docs.docker.com/compose; version 1.25.5), while the command line client is distributed using the Python Package Index (PyPi; https://pypi.org). Furthermore, Isabl's metadata infrastructure is decoupled and agnostic of compute and data storage environments (e.g. local, cluster, cloud). This functionality separates dependencies, fosters interoperability across data processing environments, and ensures that metadata is accessible even when the data is no longer available (FAIR A2 [13]). Isabl API is documented with ReDoc (https://platform.isabl.io/redoc/; https://github.com/Rebilly/ReDoc version 2.0.0; FAIR I3 [13]) following OpenAPI specifications (https://www.openapis.org; FAIR I2 [13]; FAIR R1.2 [13]).

Furthermore, Isabl is a framework. This means that Isabl API and Isabl CLI are installed as external dependencies, guaranteeing compatibility with future upgrades. As a result, end-users don't have to alter Isabl's source code to extend or modify the platform functionality (i.e. adding support for diverse data modalities such as imaging, radiology etc.).

### Isabl API and CLI

Isabl's backend (Isabl API) was written in Python (http://www.python.org; version 3.7) using the Django (https://djangoproject.com; version 2.1.3) web framework as a reusable application (https://docs.djangoproject.com/en/2.1/ref/applications) so that users can install it as a dependency without the need to fork out from source code in order to extend their services. The django package was bootstrapped using Cookiecutter (https://github.com/audreyr/cookiecutter; version 1.7.2) from Cookiecutter Django Package (https://github.com/pydanny/cookiecutter-djangopackage; version 2.0.2). PostgreSQL (https://www.postgresql.org; version 10.1) was used to deliver Isabl's database. Django Taggit (https://github.com/alex/django-taggit; version 0.23.0) was used to support tagging capabilities. The RESTful API was implemented using Django REST Framework (DRF; www.django-rest-framework.org; version 3.8.2). The RESTful API swagger documentation was made available using drf-yasg (https://github.com/axnsan12/drf-yasg; version 1.16.1). Django Filter, DRF Filters, and DRF Query Fields were used to support advanced API filtering (https://github.com/philipn/django-rest-framework-filters version 1.0.0, https://github.com/carltongibson/django-filter version 2.0.0, and https://github.com/wimglenn/djangorestframework-queryfields version 1.0.0, respectively). RESTful API authentication was supported by Django Rest Auth (https://github.com/Tivix/django-rest-auth; version 0.9.2). Django Reversion was used to provide metadata version control (https://github.com/etianen/django-reversion; version 4.0.4). Excel files processing was conducted using XlsxWriter (https://github.com/jmcnamara/XlsxWriter; version 0.9.8). Isabl CLI was also bootstrapped with Cookiecutter from Cookiecutter PyPackage (https://github.com/audreyr/cookiecutter-pypackage; version 0.1.1). Command line functionalities were provided by Click (https://github.com/pallets/click; version 7.0) while mechanisms to conduct HTTP operations were brought by Requests (http://docs.python-requests.org; version 2.23.0).

### Isabl web

The user interface was developed as an interactive single-page application using Vue (https://vuejs.org; version 2.5.16), a javascript web development framework. It’s delivered as a node (https://nodejs.org) package through NPM (https://www.npmjs.com), so it can be consumed by any developer in it’s own web page. Currently, it comes by default when Isabl's cookiecutter (https://github.com/isabl-io/cookiecutter-api) is used to generate a ready-to-go sample django project. As a Vue package, Isabl Web uses vue’s most common libraries, such as vue-cli for local development (https://cli.vuejs.org; version 3.3.0), vuex for state and data management (https://vuex.vuejs.org; version 3.0.1), vue-router (https://router.vuejs.org; version 3.0.1) for page browsing, vuetify (https://vuetifyjs.com; version 1.1.9) as a styled-components framework based on google’s Material Design (https://material.io/design), and several other open-source packages for specific desired features within the interface: vue-gallery, vue-json-excel, vue-upload-component, vue-clipboard, vuex-router-sync, v-hotkey, among others, all available from the NPM registry. Moment (https://momentjs.com; version 2.22.2) is used to parse dates and times, and D3 (https://d3js.org; version 5.9.7) to create interactive components to show results and reports, such as the Individual-Experiment-Sample tree. Within development, a handful amount of libraries are used to boost capabilities of javascript, HTML and CSS; babel (https://babeljs.io; version 7.0.0) allows to use the latest ES6 features by compiling modern javascript into browser-compatible one, sass (https://sass-lang.com) extends CSS and facilitate its use, webpack (https://webpack.js.org; version 4.0.0) is used to create a single-page bundle to publish, and finally jest (https://jestjs.io; version 23.0.1) and cypress (https://www.cypress.io; version 1.10.1) are used to create unit and end-to-end tests, without extensive configuration.

### Continuous integration, testing, and documentation

The RESTful API, database, and Web App were orchestrated using Docker Compose (https://docs.docker.com/compose; version 1.25.5) while all dependencies were resolved using Docker (https://www.docker.com; version 19.03.8). The Isabl CLI dependencies were limited to python libraries ensuring that the project was pip-installable (https://pypi.org/project/pip; version 20.1). All code repositories were Continuously Integrated (CI) using Travis CI (https://travis-ci.org), small code changes were merged frequently—rather than large changes at the end of development cycles. This was ensured by automatically running tests in the cloud upon every new code change. Testing was conducted and implemented with Pytest (https://docs.pytest.org; version 3.7.4) and tox (https://pypi.org/project/tox; version 2.9.1). Moreover, + 90% test coverage was guaranteed and automatically evaluated on the cloud using Coverage.py (https://pypi.org/project/coverage; version 4.4.2) and Codecov (https://codecov.io) for python projects. Extensive checks on documentation and code quality standards were ensured using ESLint (https://eslint.org; version 4.19.1), Pylint (https://www.pylint.org; version 1.8.1), and Pydocstyle (https://pypi.org/project/pydocstyle; version 2.1.1). Production code was homogenized and formatted with Black (https://github.com/ambv/black; version 18.9b0) and Prettier (https://prettier.io; version 1.12.1). Continuous Deployment (CD) of the components to their respective package managers and hosting sites was automatically conducted upon new releases. Both Isabl API and Isabl CLI were deployed to the Python Package Index (PyPi; https://pypi.org), while Isabl Web was deployed to npm (https://www.npmjs.com). Isabl's documentation is stored on GitHub (https://github.com/isabl-io/docs) and can be browsed at https://docs.isabl.io.

### Cookiecutter toil and toil container

Cookiecutter Toil (https://github.com/papaemmelab/cookiecutter-toil) was forked from Cookiecutter PyPackage. Similarly to Isabl CLI, Toil Container (https://github.com/papaemmelab/toil_container) was bootstrapped with Cookiecutter PyPackage. The mechanisms to perform Docker (https://www.docker.com) and Singularity (https://singularity.lbl.gov; version 2.6) system calls were implemented as adaptations of Toil's apiDockerCall (https://github.com/DataBiosphere/toil/blob/d23f7ec46d2006c136a2a5b4e57eadfb44a606b7/src/toil/lib/docker.py#L199) and Toil-vg's singularityCall (https://github.com/vgteam/toil-vg/blob/48645cbf9c1e36c73abf2f731b3f06607185a5e9/src/toil_vg/singularity.py#L22). Toil Container was developed following the same standards and testing procedures of Isabl. Alike Isabl CLI, Cookiecutter Toil and Toil Container were deployed to PyPi.

## Supplementary information

**Additional file 1.** Supplementary figures and notes related to this manuscript.

## Data Availability

Isabl Platform is free for academic and non-profit institutions, source code can be requested at licenses@isabl.io.

## Abbreviations

AIMS: Analysis information management system
WGS: Whole genome sequencing
NGS: Next generation sequencing
CLI: Command line interface
SPA: Single page application
HPC: High performance computing
AI: Artificial intelligence
UUID: Universally unique identifier
SDK: Software development kit
WMS: Workflow management system
