# Supplementary material for: Isabl Platform, a digital biobank for processing multimodal patient data
Source: BMC Bioinformatics. 2020 Nov 30;21:549. doi: 10.1186/s12859-020-03879-7 (PMC7708092; doi:10.1186/s12859-020-03879-7)
Supplement: Supplementary file 1 — Additional file 1. Supplementary figures and notes related to this manuscript. [file 12859_2020_3879_MOESM1_ESM.docx]

# Supplementary Information

[Supplementary Figure 1: Database](#_r6rm7p96ups5) **2**

[Supplementary Figure 2: System ID](#_4nlbiquebtxr) **3**

[Supplementary Figure 3: Analysis Panel](#_v6j7ceou9i07) **5**

[Supplementary Figure 4: Project Panel](#_n70xz63yll6f) **7**

[Supplementary Figure 5: Samples Panel](#_199o1w1z7g2a) **9**

[Supplementary Figure 6: Dashboards](#_5bimvr62svx) **11**

[Supplementary Figure 7: Data Import](#_wv696swp4ybh) **12**

[Supplementary Figure 8: Application Example](#_82u8zer1e7ez) **13**

[Supplementary Figure 9: Automations](#_h78ro62nkxdp) **15**

[Supplementary Figure 10: Cookiecutter Toil and Toil Container](#_uqdiib9c9g7z) **17**

[Supplementary Video 1: Audit Trail](#_atx4wri73quv) **19**

[Supplementary Notes 1: Platforms Comparison](#_vd2eanf4fji7) **19**

[Metadata capabilities](#_9x6wie4rnxxc) 20

[Assets management](#_cgweugv19dfn) 20

[Data processing](#_wpp0qurgpvz) 20

[Results and metadata accessibility](#_stwe2yf1v63c) 21

[Codebase status and availability](#_qaza7pepmphh) 21

[References](#_imk6h4zbr3tl) 21

### Supplementary Figure 1: Database ([download full size](https://user-images.githubusercontent.com/8843150/73288831-b3438500-41c9-11ea-83da-d4a573179f41.png))


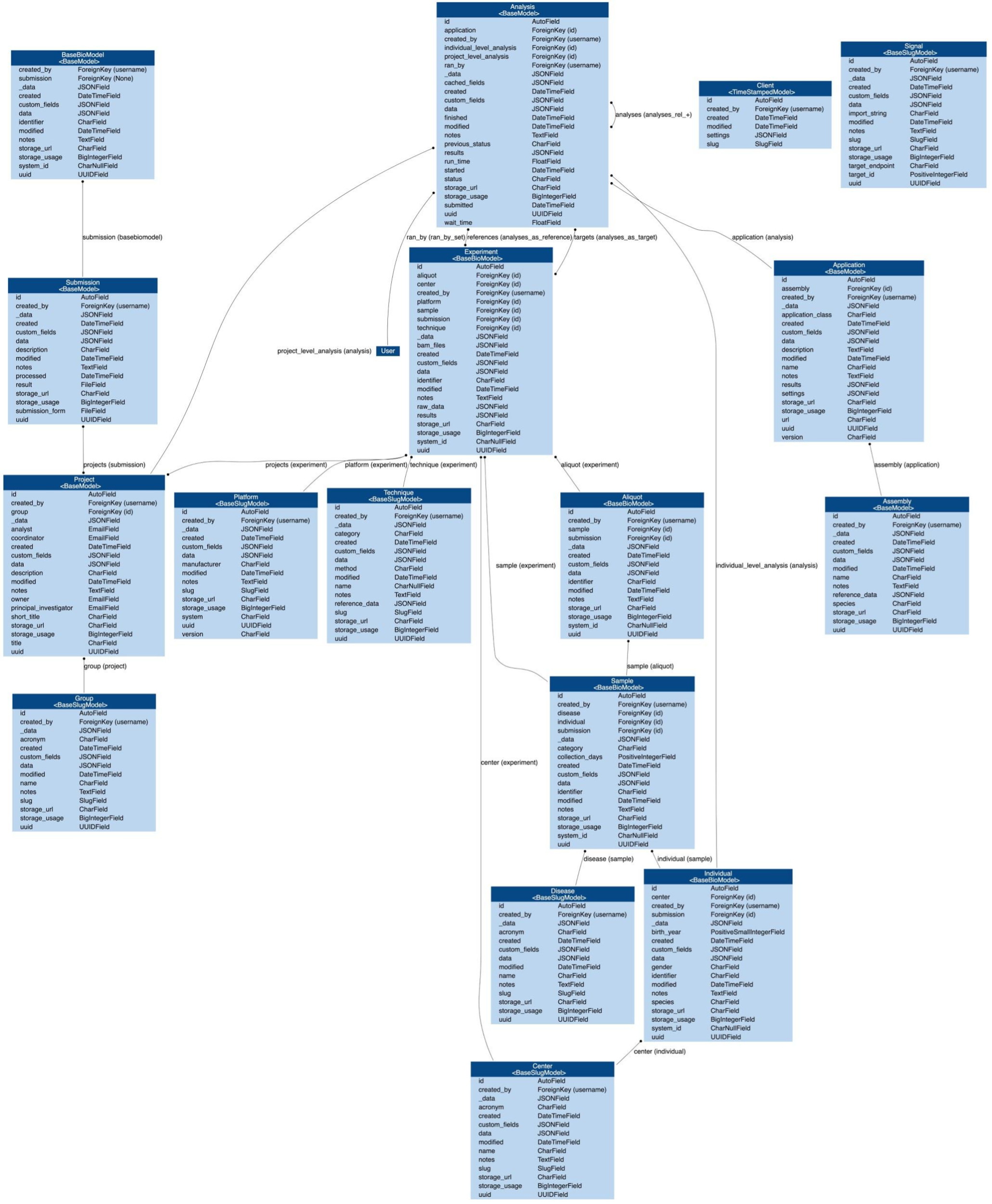


**Supplementary Fig. 1:** A complete map of Isabl DB relational model and fields. Each endpoint in Isabl API includes filters that can traverse these relationships.

### Supplementary Figure 2: System ID ([download full size](https://docs.google.com/drawings/d/e/2PACX-1vSfEIOMbODpSWvwtTGjVxwwf0xekeBIaM3TfkfpOnzAScw9i7w-7xoafgaCNeB-UUuB5l90IG_q6-PZ/pub?w=1214&h=572))


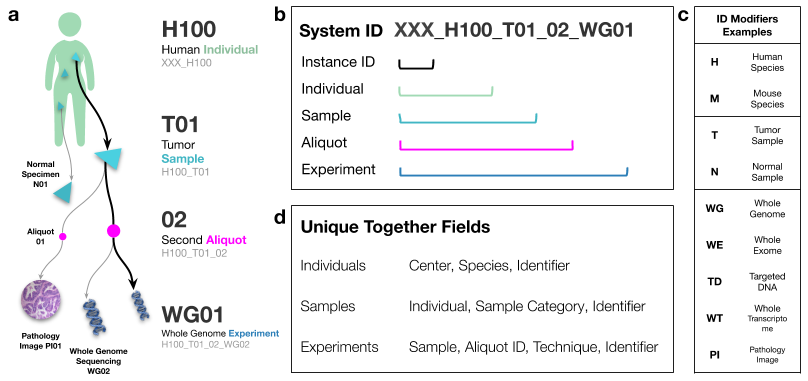


**Supplementary Fig. 2:** A user friendly identifier to represent individuals, samples, and experiments. **a** The Isabl ID implementation aims to provide a representation of the data generation process while providing a glimpse on key metadata attributes, such as the individual species, the sample category, and the technique used to generate the experimental data. **b** The first component of the identifier is a customisable *instance ID*, it is used to differentiate identifiers generated by different Isabl instances potentially running in the same institute. **c** Metadata modifiers are embedded in the ID and can be extended. For example, *H100_T01_02_WG01* identifies the first *whole genome* experiment generated from the second *aliquot* (i.e. biological replicate) of *patient 100’s* first *tumor sample*. The counters (i.e. T01, WG02, etc.) are automatically added and reflect prior samples registration as well as key metadata characteristics. **d** Some fields are expected to be *unique together* across all instances within the infrastructure (see <https://docs.djangoproject.com/en/2.1/ref/models/options/#unique-together>). For example, two individuals coming from the same center and having the same species and identifier, are considered as the same individual. These constraints prevent duplicates and enable system wide associations between individuals, samples, aliquots, and experiments (i.e. across projects).

### Supplementary Figure 3: Analysis Panel ([download full size](https://docs.google.com/drawings/d/e/2PACX-1vR6jI-FMk97PSrFnJEFuBC8aoqjCjPMALLrzL9AvcIi_50HexoSOuQDtE3b1XGul5eVhRPE0Q78uASe/pub?w=2769&h=3552))


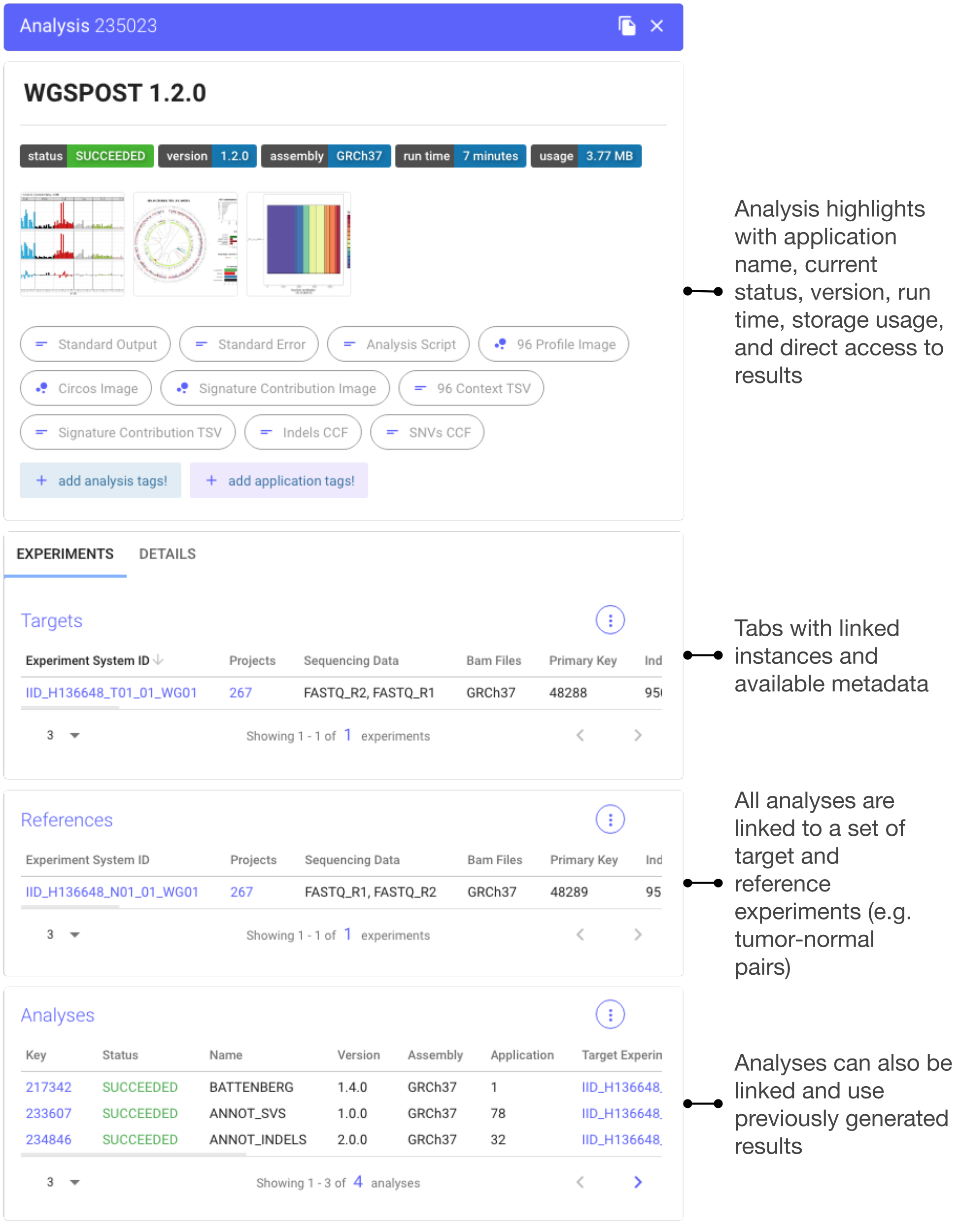


**Supplementary Fig. 3**: Isabl applications standardize the deployment of tools and pipelines given available experimental metadata. Application results are stored as analyses. The analysis panel informs about the application version, current status, run time, and storage usage. Additionally, users can directly access a list of selected results with support for multiple formats. The uniqueness of an analysis is determined by the list of target and reference experiments used (e.g. tumor-normal pairs). Applications can compute on previously generated results, these are also linked and displayed in the analysis panel. Linked analyses and experiments are displayed using *dynamic tables,* interactive data frames directly wired to Isabl API with support for searching, sorting, and column-specific filtering. Detail views are retrieved by clicking on any *linked* *identifier* within these tables. Dynamic tables are used throughout the entire web application.

### Supplementary Figure 4: Project Panel ([download full size](https://docs.google.com/drawings/d/e/2PACX-1vR3RxxkX6UGDx16VFcJ9De1M0VOcrA9ReuzZt0dEyGjtA51yxUOSMWJ_Qt3A7XgCGOoAs91VvXqTa9G/pub?w=3008&h=3632))


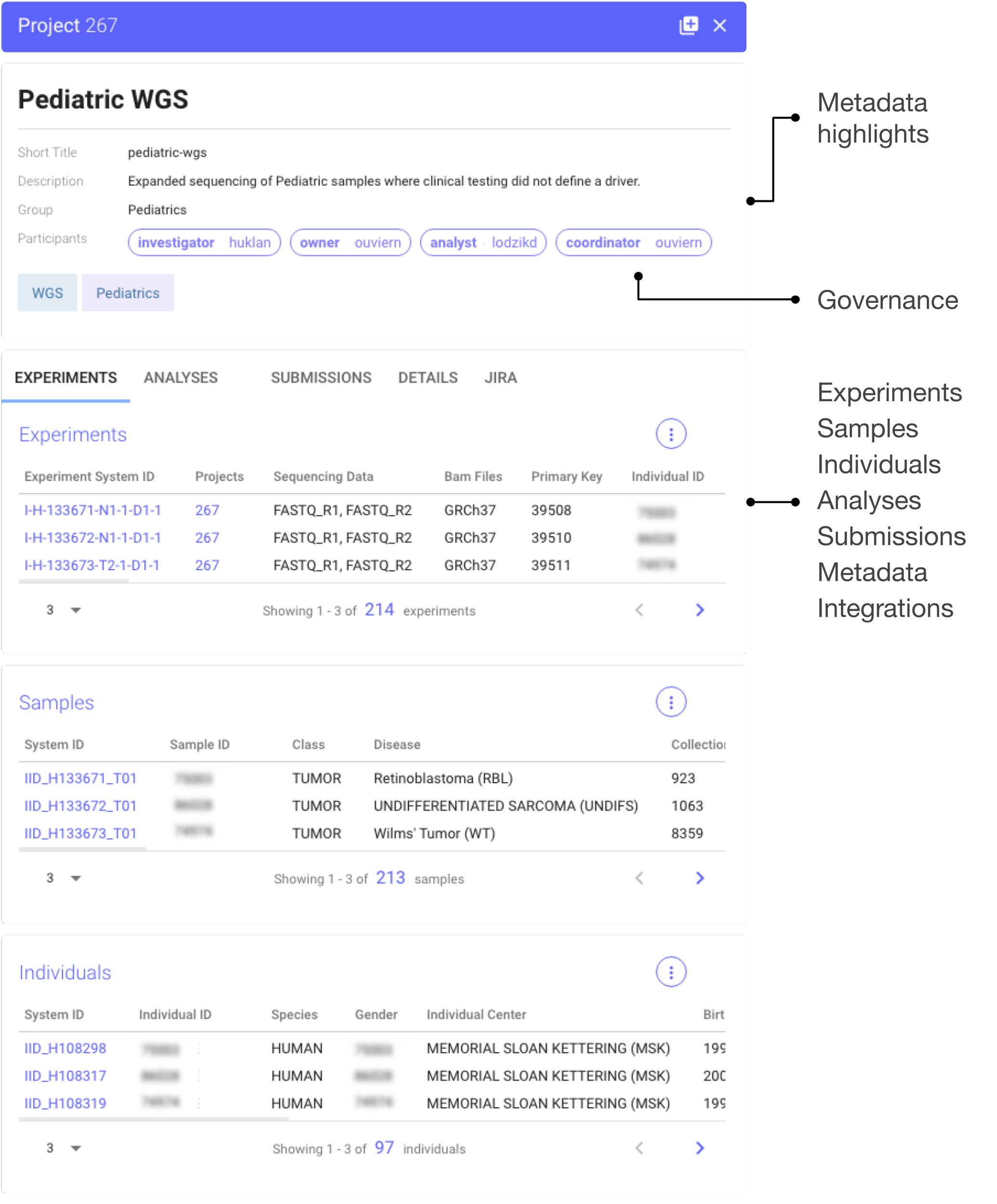


**Supplementary Fig. 4:** The project panel offers direct access to all assets related to a given study, from individuals, samples, experiments, analyses, metadata batch submissions, and metadata, all available in one place. Stakeholders, particularly the principal investigator, project owner (e.g. postdoc), an analyst, and if required, a project manager or coordinator, are displayed. Isabl projects can be integrated with JIRA ([http://jira.com](http://jira.com/)), a tasks management tool popular across software development and data science teams.

### Supplementary Figure 5: Samples Panel ([download full size](https://docs.google.com/drawings/d/e/2PACX-1vQd_I-6cj2FBwViQBnpOcoH69Ag8DRSMKrULgPOfwxJhLbPcODnLApoLVW8mB3ZklmNIHWU4CibfJXI/pub?w=1734&h=2430))


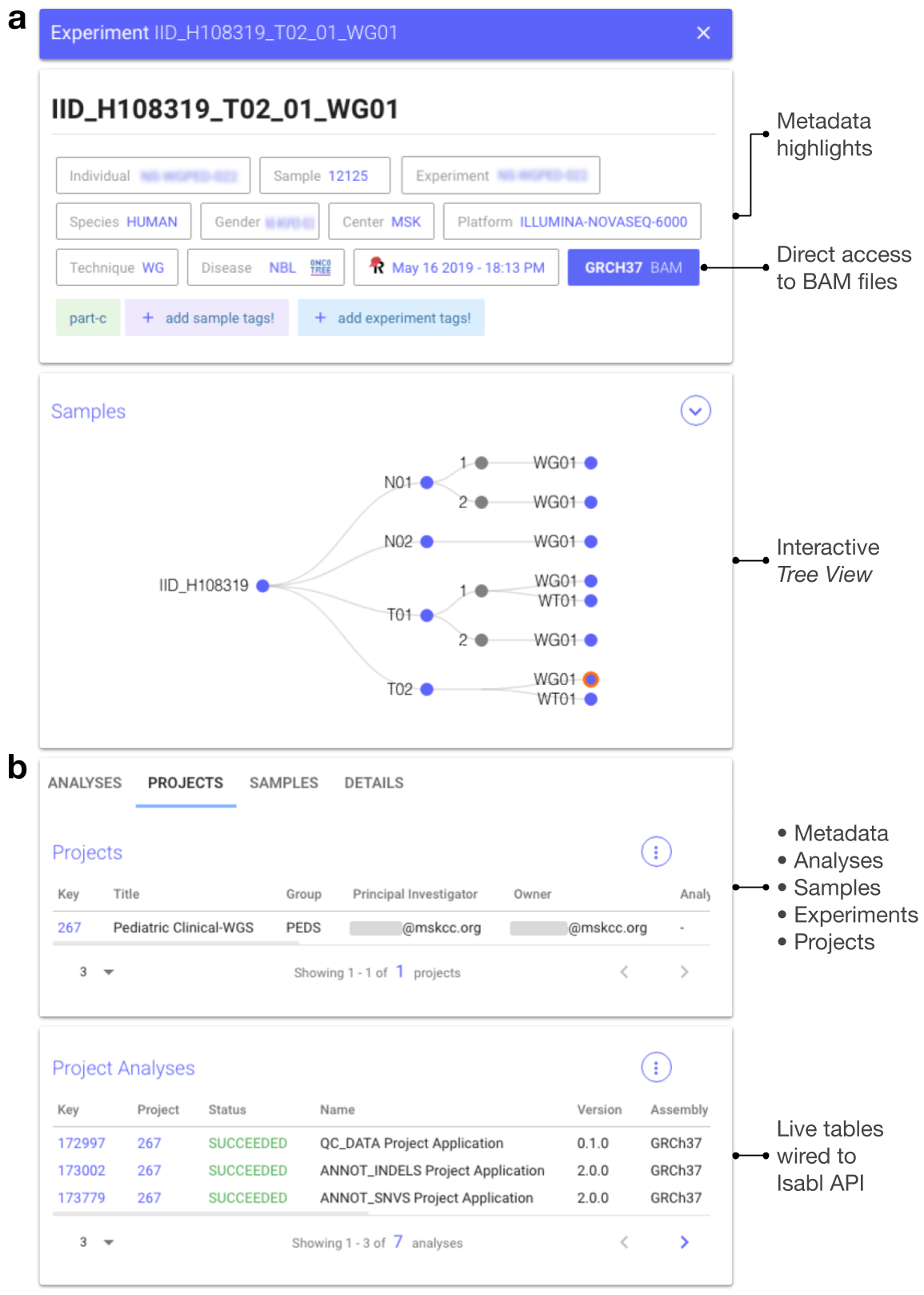


**Supplementary Fig. 5:** Isabl is an individual-centric infrastructure. **a** Isabl's samples panel provides a birds-eye view of all available assets for a given individual. The top card provides key highlights of relevant metadata for the currently selected instance. For example, the figure illustrates the detail view of a Whole Genome Sequencing experiment conducted on the second tumor of a patient. The *GRCh37 BAM* button gives users the ability to access aligned data in real time using IGV.js ([https://github.com/igvteam/igv.js](https://github.com/igvteam/igv.js/)). Custom highlights can be added, for example here we display the last date when metadata was synced from a clinical database (REDCap). The samples card, or *Tree View*, enables navigation across all instances generated on a given individual. **b** Several tabs are provided with *dynamic tables* with access to linked analyses, projects, samples and experiments, as well as a full list of all metadata attributes available in Isabl DB.

### Supplementary Figure 6: Dashboards ([download full size](https://docs.google.com/drawings/d/e/2PACX-1vRAeYSybbYF8ZXrFv8Yy1fOwTZFq2yF0zuScuRkcrvWhXY32CON3ArpYxlfAlx6o0KJWT319wmw84jV/pub?w=7392&h=5433))


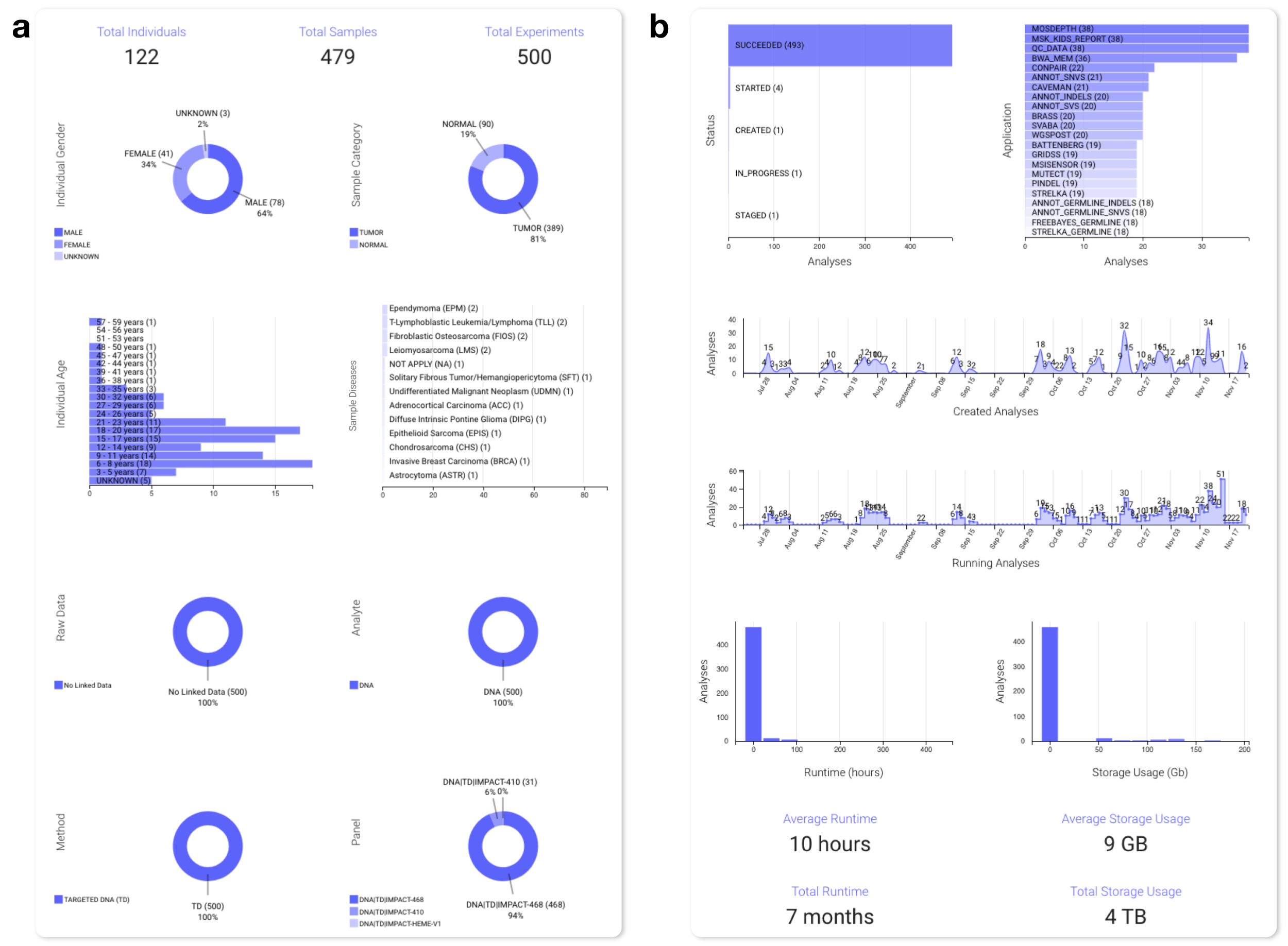


**Supplementary Fig. 6:** Isabl Web includes interactive dashboards to explore metadata and access results. **a** The samples dashboard describes individuals by age and gender, samples by category and disease, and experiments by data linkage, analyte, sequencing method, and technique name. **b** The analysis dashboard allows filtering across status, applications, created and execution dates, and distributions of runtime and storage usage.

### Supplementary Figure 7: Data Import ([download full size](https://docs.google.com/drawings/d/e/2PACX-1vTXjWyPRNRKJaj5R5_dZgQWkVEJ3HjOFdMP2XIir9cFv0pMvXRC7fuzN-xyAAiFr6SWA70hq8yqBxBI/pub?w=1124&h=788))


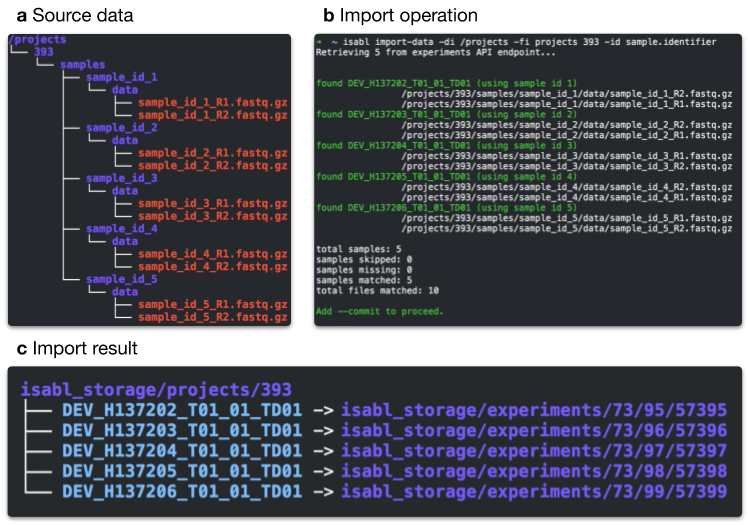


**Supplementary Fig. 7:** Isabl CLI supports automated data import from data deposition directories. To do so, the client explores these directories recursively matching digital assets (e.g. genomic, imaging) with identifiers registered in the database. For example, given a directory (**a** Source Data), the client can be instructed to explore the non-Isabl */projects* directory, retrieving only samples from *Project 393*, and match files using *Sample Identifiers* (**b** Import Operation). Upon *commit*, Isabl CLI proceeds to move (or symlink) matched files into scalable directory structures (**c** Import Results). The experiment's data path is created by hashing the four last digits of its primary key. For instance, data for Experiment 57395 will be stored at *{data-directory}/experiments/73/95/57395/*. This hashing approach is also used to store analyses outputs and ensures a maximum of 1000 subdirectories in any folder at a worst case scenario of 10 million instances.

### Supplementary Figure 8: Application Example

###
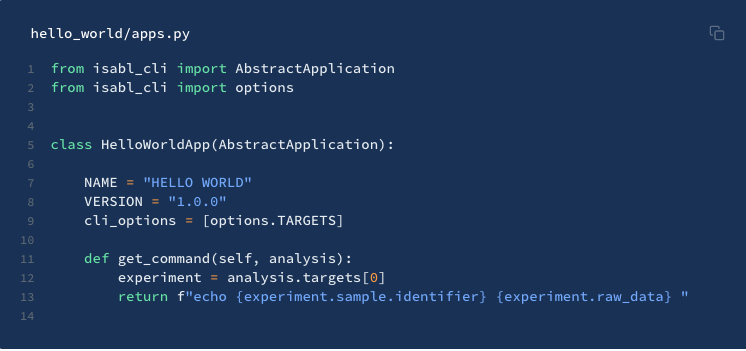


**Supplementary Fig. 8:** Isabl applications are configured using python classes, inheriting from an *abstract application* which already solves most of the heavy lifting. Developers have to implement a set of methods and attributes in order to wrap data processing pipelines and tools into an application. Note that the ultimate objective is to build and validate commands given metadata stored in Isabl DB that will be deployed to a computing environment (e.g. HPC, Cloud, Local). The example in the figure presents a *hello world* application that simply echoes some attributes of the experiment linked to the analysis (i.e. the sample identifier and associated raw data). By setting *cli_options* (i.e. command line options), Isabl facilitates the execution of this command across any experiment in the database, storing results in their own analysis output directory. A full guide on writing Isabl applications can be found at <https://docs.isabl.io/writing-applications>.

### Supplementary Figure 9: Automations ([download full size](https://docs.google.com/drawings/d/e/2PACX-1vRHG1UJdtzi-jqHuvkjdpnpzgysEDVEM12uM_t4G3AA225oxVdC-Sp6WzqsZwiTG_RYIsQBKnvoySAl/pub?w=1296&h=1584))


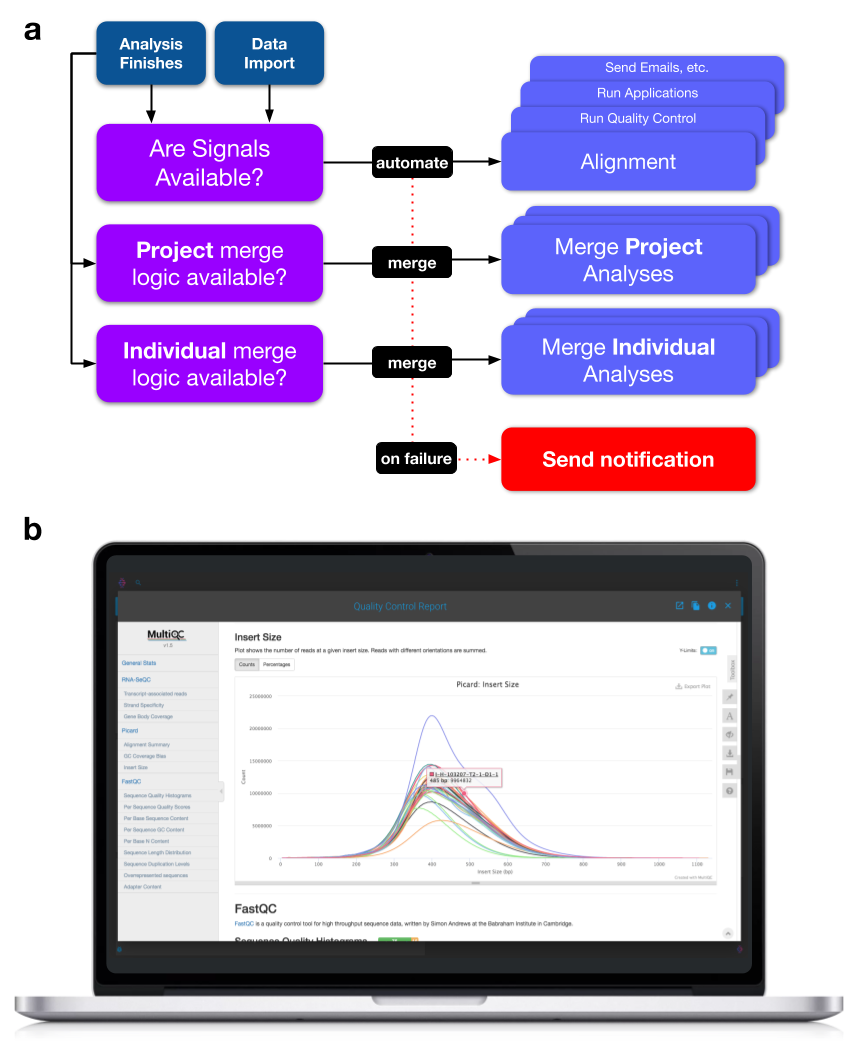


**Supplementary Fig. 9:** Isabl is built for automation and autonomy. **a** Users can define *signals (i.e. events)* —python functions that will be executed upon data import or analyses status change. These signals encode logic for operational automations, such as alignment, quality control, dependency graphs, and any other required action. Beyond *signals*, Isabl applications can produce auto-merge analyses at a project and individual level. For example, users may want to merge variants whenever new results are available for a given project, or update quality control reports when a new sample is added to an individual. **b** An example of a project-level quality control report generated using MultiQC ([http://multiqc.info](http://multiqc.info/)) after merging independently generated metrics for each experiment in the project, rendered in Isabl Web.

### Supplementary Figure 10: Cookiecutter Toil and Toil Container ([full size](https://docs.google.com/drawings/d/e/2PACX-1vT8_ifYzMvXChAw2moj9O4wAmRNJZuUulCThbSPIp5E8MKS92cG186TyaRgxo0H6TLc1NoPPz_3fEnw/pub?w=3432&h=3696))


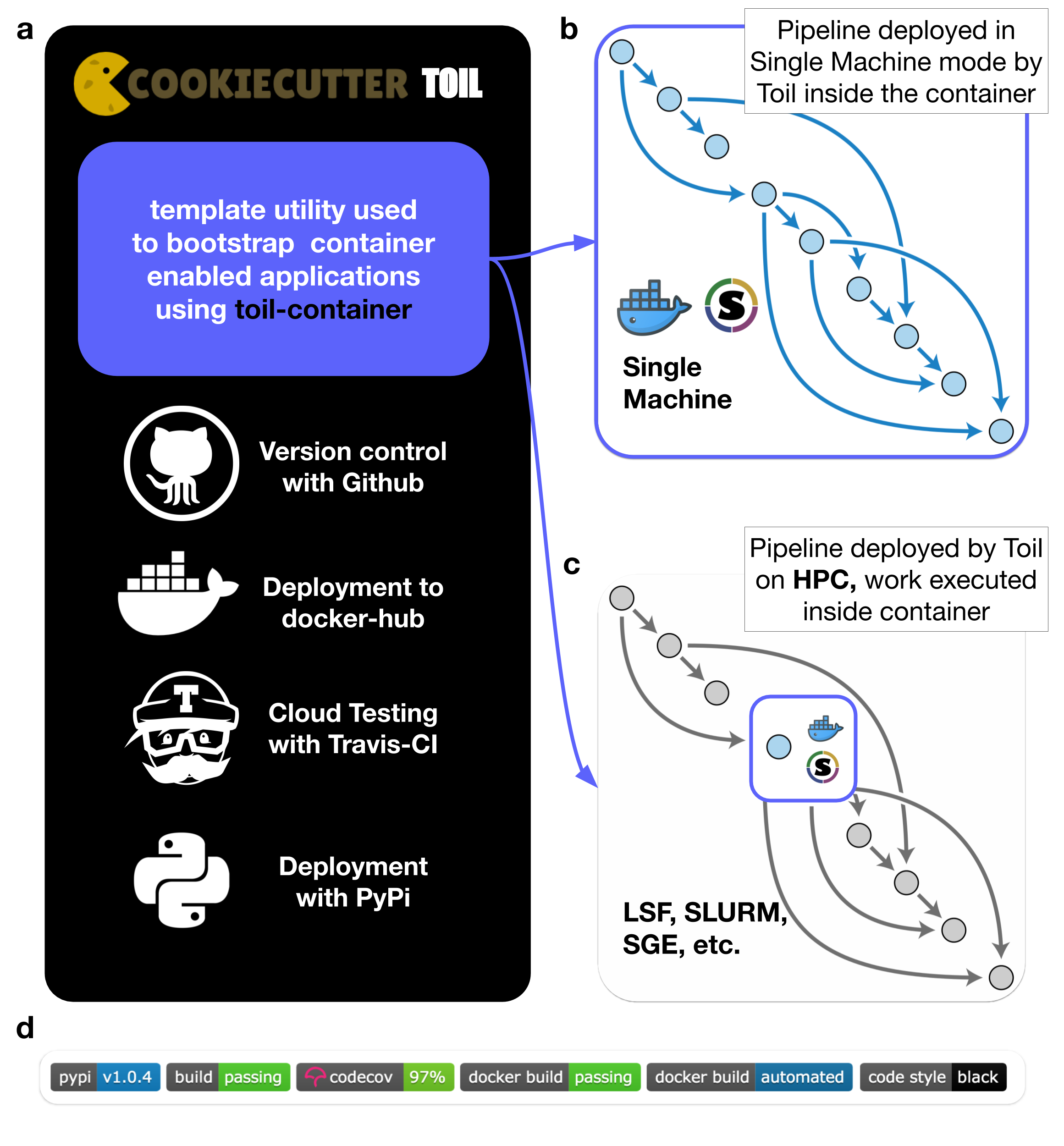


**Supplementary Fig. 10: a** Cookiecutter Toil ([https://github.com/papaemmelab/cookiecutter-toil](https://github.com/leukgen/cookiecutter-toil)) is a templating utility that creates tools with built-in software development best practices: 1. a version-controlled Toil pipeline template with 100% test coverage (click is supported for simpler tools; <http://click.pocoo.org>); 2. a base Docker configuration with Singularity usage documentation; 3. a cloud testing configuration that guarantees correct containerization and coding standards; and 4. automated deployment to the python package index and Docker Hub ([https://hub.docker.com](https://hub.docker.com/)) upon new github releases. Cookiecutter Toil pipelines include extensive contributing guidelines and a code of conduct ([https://www.contributor-covenant.org](https://www.contributor-covenant.org/)). An example of a project generated with the latest version of Cookiecutter Toil is available at [https://github.com/papaemmelab/toil_example](https://github.com/leukgen/toil_example). **b** Toil Container enables Toil class-based pipelines, i.e. Direct Acyclic Graphs (DAGs; <https://github.com/DataBiosphere/toil>), to perform containerized system calls with both Docker and Singularity without source code changes. Toil Container pipelines can be executed in *single-machine* mode inside the container, which is simple but limited in computational resources. **c** Alternatively, pipelines can be installed within HPC environments to achieve massive parallelization across multiple computing nodes. In this way, the execution graph is deployed by Toil in *batch-system* mode, while each job performs containerized system calls. **c** An example of best-practice badges that will be included in repositories initialized with Cookiecutter Toil (<https://github.com/badges/shields>).

### Supplementary Video 1: Audit Trail ([video link](https://www.youtube.com/watch?v=L1JhVqZ3oBY&feature=youtu.be))

[
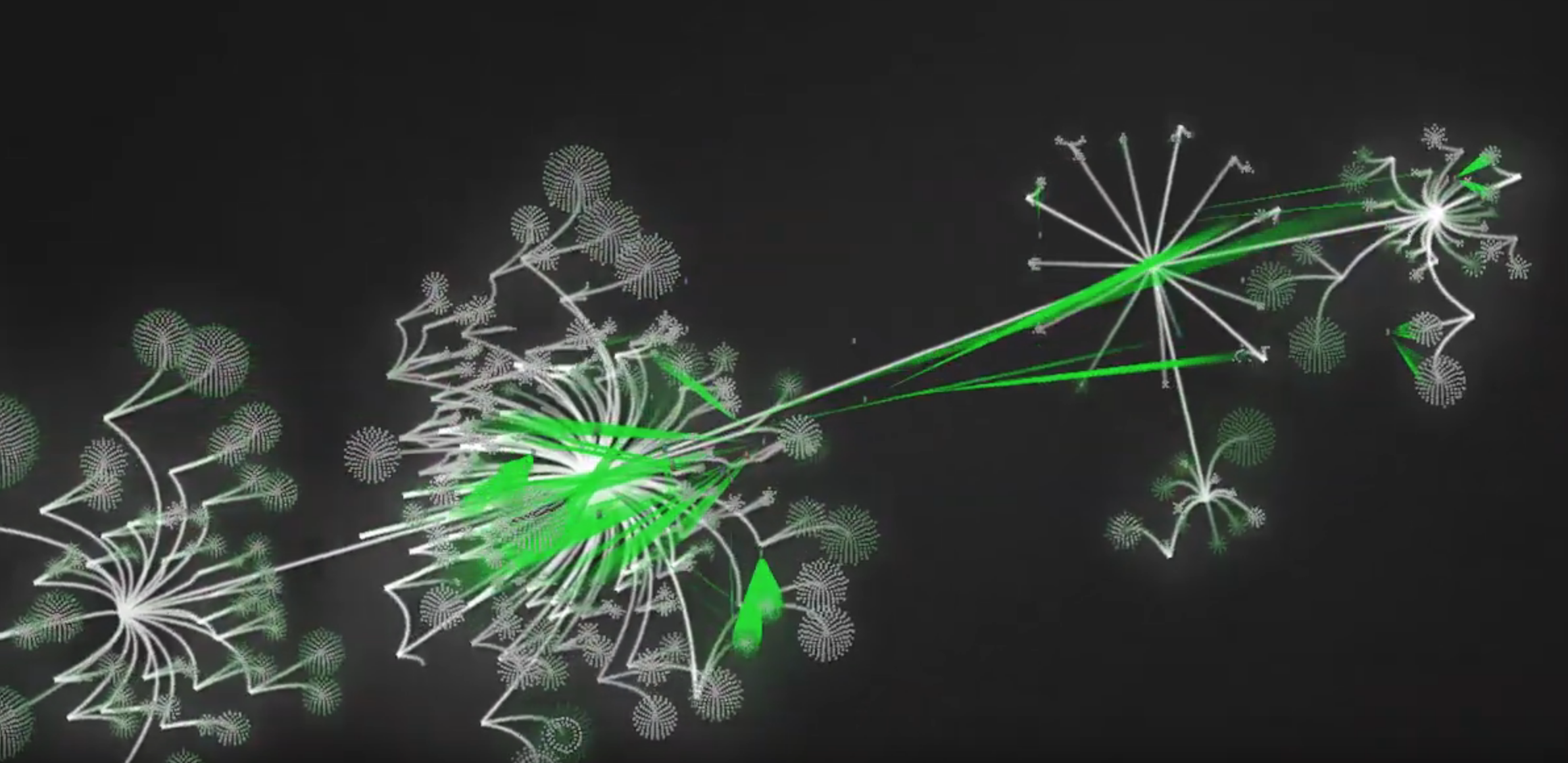
](https://www.youtube.com/watch?v=4F_XhHWTPlw)

**Supplementary Video 1:** More than 60 users generating meta(data) across projects showcases the complexity of information governance and provenance. The *tree-like* graph generated in the video has 4 levels: (1) group (i.e. department) to which a given project belongs to; (2) project identifier; (3) center from which the individual was referred from; (4) individual gender; and (5) one node per experiment generated on each individual.

### Supplementary Notes 1: Platforms Comparison

The following five sections highlight differences in design paradigms between Isabl and the frameworks listed in Table 1.

#### Metadata capabilities

All platforms are built with relational databases. Isabl and SeqWare include a RESTful API, and only Isabl provides a searchable single-page swagger documentation. Isabl and SeqWare offer the most mechanisms for metadata ingestion and integration with web forms, excel batch submissions, CLI methods, and RESTful API operations. Isabl, GMS, and SeqWare all share an individual-centric relational model with support for individuals, samples, and experiments. In contrast, QuickNGS and HTS-flow follow a denormalized approach for experiments annotation.

#### Assets management

Isabl provides automated import logic of experimental data into a scalable directory structure limited to the CLI. SeqWare has web *and* CLI data import mechanisms with AWS support. QuickNGS relies on manually created symbolic links, while HTS-flow features LIMS integration and supports external data. Data can be imported into the GMS on a one by one basis using the CLI. Additionally, Isabl and GMS have built-in protocols to import and database auxiliary reference data into the system.

#### Data processing

Isabl is agnostic to pipeline implementation, compute environments, and importantly does not dictate workflow management specifications. Conversely, other platforms require adherence to specific workflow management systems and pipeline development paradigms[^1–3^](https://paperpile.com/c/k2azno/EqwPW+Td9wB+uKFEs). While Isabl does not come with pre-built tools, we have developed and deployed a framework for end-users to easily incorporate and share applications. This design makes Isabl lightweight while providing end-users complete autonomy with regard to their operational workflows, implementation of analysis tools, and choice of compute infrastructure.

#### Results and metadata accessibility

Both Isabl and GMS offer comprehensive Software Development Kits (SDKs). QuickNGS limits data provisioning to web downloads, while the rest of the platforms also provide CLI tools and direct file system access. Although having a web portal for status monitoring, results retrieval, and metadata management is common across the systems, only Isabl offers a SPA.

#### Codebase status and availability

Isabl is available on an academic licence in a plug-and-play and light-weight configuration powered by Docker Compose and the Python Package Index (PyPi), and has extensive online documentation which is version controlled. To showcase this functionality we developed “*10 Minutes to Isabl”* (<https://docs.isabl.io/quick-start>), a tutorial that guides end-users with a personal computer through platform installation, project registration, data import, application execution, and results retrieval. Other platforms, however, come with pre-built applications[^1,2,4^](https://paperpile.com/c/k2azno/y42Py+EqwPW+Td9wB) and pre-installed external software[^2,4^](https://paperpile.com/c/k2azno/Td9wB+y42Py) resulting in heavy installation bundles and virtual machines.

#### References

1. [O’Connor, B. D., Merriman, B. & Nelson, S. F. SeqWare Query Engine: storing and searching sequence data in the cloud. *BMC Bioinformatics* **11 Suppl 12**, S2 (2010).](http://paperpile.com/b/k2azno/EqwPW)

2. [Griffith, M. *et al.* Genome Modeling System: A Knowledge Management Platform for Genomics. *PLoS Comput. Biol.* **11**, e1004274 (2015).](http://paperpile.com/b/k2azno/Td9wB)

3. [Bianchi, V. *et al.* Integrated Systems for NGS Data Management and Analysis: Open Issues and Available Solutions. *Front. Genet.* **7**, 75 (2016).](http://paperpile.com/b/k2azno/uKFEs)

4. [Wagle, P., Nikolić, M. & Frommolt, P. QuickNGS elevates Next-Generation Sequencing data analysis to a new level of automation. *BMC Genomics* **16**, 487 (2015).](http://paperpile.com/b/k2azno/y42Py)
